# Revascularización Coronaria en Enfermedad de Kawasaki

**DOI:** 10.47487/apcyccv.v1i2.58

**Published:** 2020-06-29

**Authors:** Carolina Cabrera-Cerna, Gabriela Bustillos-García, Juan Calderón-Colmenero, José García-Montes, Ana Trujeque-Ruiz, Édgar Ramírez-Marroquín

**Affiliations:** 1 Médico residente de Cardiología Pediátrica. Instituto Nacional de Cardiología Ignacio Chávez, México. Médico residente de Cardiología Pediátrica. Instituto Nacional de Cardiología Ignacio Chávez México; 2 Departamento de Cardiología Pediátrica. Instituto Nacional de Cardiología Ignacio Chávez, México. Departamento de Cardiología Pediátrica Instituto Nacional de Cardiología Ignacio Chávez México; 3 Departamento de Cirugía Cardio-torácica Adultos y Pediátrica. Instituto Nacional de Cardiología Ignacio Chávez, México. Departamento de Cirugía Cardio-torácica Adultos y Pediátrica Instituto Nacional de Cardiología Ignacio Chávez México

**Keywords:** enfermedad de Kawasaki, aneurisma coronario, revascularización miocárdica, Kawasaki’s disease, coronary aneurysm, myocardial revascularization

## Abstract

La enfermedad de Kawasaki (EK) ha sobrepasado en muchos países a la fiebre reumática como primera causa de cardiopatía adquirida en la edad pediátrica, siendo su secuela más grave los aneurismas coronarios. Presentamos un caso de revascularización quirúrgica exitosa en un paciente de 5 años de edad, con lesión trivascular coronaria secundaria a EK, a quien se le realizó una derivación coronaria con anastomosis de la arteria mamaria interna izquierda a la descendente anterior, la arteria mamaria derecha a la obtusa marginal y la arteria radial a la descendente posterior. Un seguimiento estrecho y la decisión oportuna de la cirugía evitarán sucesos desfavorables como incapacidad permanente del paciente, disfunción ventricular, arritmias, infarto al miocardio y muerte súbita.

## Abstract (en)

Kawasaki´s disease (KD) has surpassed rheumatic fever in many countries as the first cause of acquired heart disease in pediatric age, being the coronary aneurism its worst complication. We present a case of successful revascularization in a 5-year-old patient, with three-vessel coronary disease secondary to KD, who underwent a coronary bypass with anastomosis from the left internal mammary artery to the anterior descending artery, the right mammary artery to the marginal obtuse artery and the radial artery to the posterior descending artery. Close follow-up and choosing the right time to undergo surgery will prevent adverse events like permanent disability of the patient, ventricular dysfunction, arrhythmias, global infarction and sudden death.

## Introducción

La enfermedad de Kawasaki (EK), se manifiesta como una vasculitis febril aguda de carácter autolimitado y de causa desconocida que afecta predominantemente a niños menores de 5 años.^1^ Fue descrita por primera vez en 1967 en Japón; ^(^^2^ y poco tiempo después, tras estudios anatomo-patológicos, se reportaron formaciones aneurismáticas en las arterias coronarias, secundarias a los cambios inflamatorios. ^(^^3^^,^^4^ De acuerdo a la evolución, durante la fase aguda hasta la fase de convalecencia, podría suceder la ruptura de estos aneurismas coronarios, trombosis o cambios proliferativos que lleven a la disminución de la luz del vaso con posterior estenosis, llevando a isquemia miocárdica, incrementando así la mortalidad de estos pacientes. ^(^^5^

Se ha descrito mayor morbimortalidad en pacientes con aneurismas coronarios gigantes, definidos como aquellos con un diámetro interno de al menos 8 mm o una puntuación Z score > 10 que no involucionan. ^(^^1^^,^^6^ Si bien la evolución de los aneurismas es la regresión espontánea de hasta un 50% de los casos en los 2 años posteriores al inicio de la enfermedad, la probabilidad de regresión es inversamente proporcional al tamaño del aneurisma. ^(^^6^ Por otro lado, el desarrollo de estenosis en la ramificación o bifurcación de la arteria coronaria ha sido descrito hasta 15 años más tarde en pacientes cuyos aneurismas coronarios presentaron un diámetro mayor a 6 mm en la etapa temprana de la enfermedad, ^(^^7^ lo que incrementa el riesgo de complicaciones como trombosis, isquemia miocárdica e infarto agudo de miocardio con mayor frecuencia en el primer año de la enfermedad; sin embargo, el riesgo de desarrollarla persiste con los años. ^(^^5^^,^^7^

La cirugía de revascularización coronaria en pediatría ha sido el abordaje utilizado por varios grupos como opción terapéutica en pacientes con EK, con buenos resultados publicados en la literatura, sobre todo en aquellos sintomáticos, con enfermedad coronaria severa (aneurismas gigantes o estenosis significativa > 50%) o evidencia de isquemia inducible y estenosis coronaria documentada angiográficamente.^8^^,^^9^ La mejora de las herramientas diagnósticas en imagen, ha permitido en las últimas décadas un diagnóstico oportuno de isquemia miocárdica y, por ende, la posibilidad de establecer un tratamiento temprano de estos pacientes.

## Descripción del Caso

Paciente pediátrico, con antecedente de diagnóstico de EK a los 9 meses de vida. En esa fecha, recibió tratamiento con inmunoglobulina y aspirina en la segunda semana de abordaje del cuadro. En el ecocardiograma se identificó lesiones coronarias importantes, por lo que se realizó cateterismo cardíaco hallándose: dilatación aneurismática fusiforme gigante del tronco de la coronaria izquierda (CI) de 4.73 mm (Z+4) x 8.10 mm (Z+3.8), origen de la descendente anterior (DA) 2.4 mm (Z+2.37), trayecto 2.83 mm (Z+3.8), origen de circunfleja 2.37 mm (Z+2) y aneurisma gigante de coronaria derecha (CD) proximal 12.36 x 17 mm (Z+16). Recibió manejo con captopril, furosemida, espironolactona y acenocumarol. 

**Figura 1:**
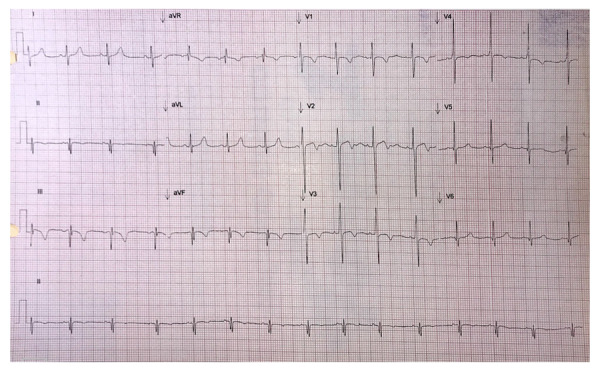
Electrocardiograma de 12 derivaciones que muestra ritmo sinusal, eje QRS -60, q profundas en II, III, aVF, onda T invertida en V1-V4, III y aVF.

**Figura 2:**
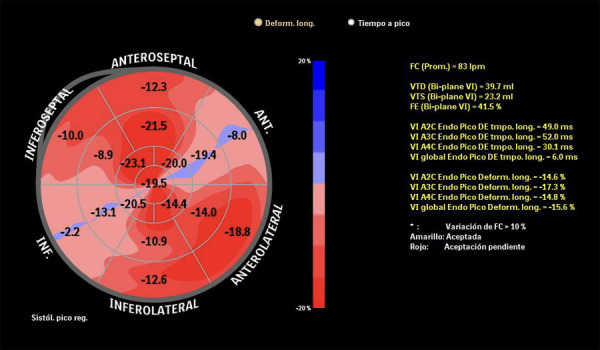
Strain Ecocardiográfico. Se muestra el bull’s eye del strain longitudinal global realizado al paciente, donde se observa alteración a nivel inferior, inferoseptal, anterior y apical, territorio de la coronaria derecha y circunfleja.

**Figura 3:**
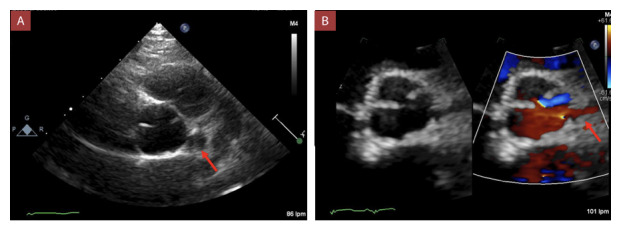
Ecocardiograma transtorácico. **A)** Prequirúrgico: Se observa un eje corto a nivel de los grandes vasos donde se puede apreciar un gran aneurisma (flecha roja). **B)** Postquirúrgico: Mismo corte que la imágen anterior, donde ya no se observa aneurisma (flecha roja).

A los 5 años de edad, en una consulta de rutina y estando asintomático, el electrocardiograma evidenció datos sugestivos de isquemia en cara inferior y sobrecarga sistólica (ritmo sinusal, eje QRS -60, Q profundas en II, III, aVF, onda T invertida en V1-V4, III y aVF. (Figura 1) El ecocardiograma transtorácico de control demostró una deformidad sistólica longitudinal global -15.6% y deformidad sistólica circunferencial global -14.4%, (Figura 2) dilatación aneurismática gigante en porción proximal de CD con masa móvil sugestiva de trombo intraluminal (15.7 mm de diámetro) y dilatación de tronco de coronaria izquierda de 7.6 x 9 mm (Z+ 6.7) y en biburfación de DA 5.1 mm (Z +5.1). (Figura 3a)

**Figura 4:**
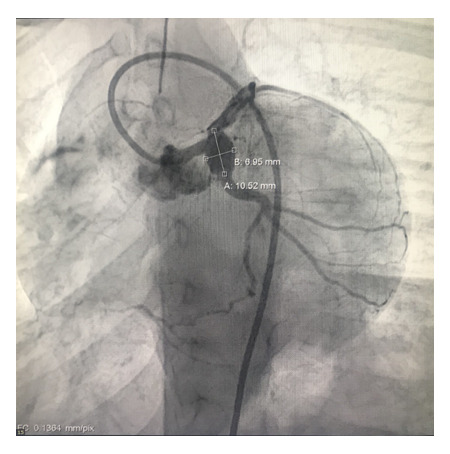
Cateterismo cardíaco. Angiografía coronaria en proyección oblicua lateral izquierda 0º y caudal 45º. Se visualiza aneurisma sacular gigante que involucra la confluencia del tronco coronario izquierdo y segmento proximal de la arteria circunfleja.

**Figura 5:**
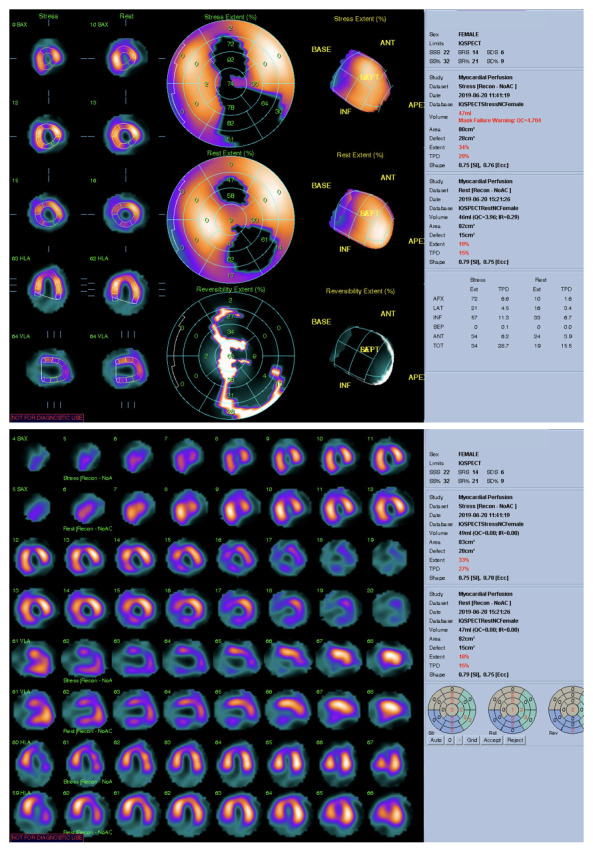
Perfusión miocárdica. **A)** En mapa polar se visualiza infarto no transmural de la pared inferior e inferolateral en toda su extensión, con isquemia ligera de tejido residual en tercio medio y basal, e isquemia moderada en su tercio apical. **B)** Imágenes tomográficas de cortes de perfusión en eje corto, vertical y horizontal del corazón se observa defecto de perfusión, con grado de isquemia al estrés de 32% y en reposo de 21%, isquemia global del 9%.

Por los hallazgos anteriores se realizó un ecocardiograma de estrés que reportó una fracción de eyección de ventrículo izquierdo (FEVI) en reposo de 45% y en estrés de 40%, hipocinesia de pared anteroseptal en reposo, e hipocinesia del ápex y pared ínfero-apical durante estrés. Por estos hallazgos, se realizó una nueva coronariografía (Figura 4) y estudio de perfusión miocárdica con el protocolo de dipiridamol y talio-201 (Figura 5a y 5b). Este último estudio informó la presencia de infarto no transmural de pared anterior en tercio apical y medio, con isquemia moderada a importante hacia el ápex en el territorio de la DA (defecto porcentual diferencial de perfusión (SD) de 6 a 9% moderada, > 9% seve-ro); infarto no transmural de la pared inferior e inferolateral en toda su extensión, con isquemia ligera de tejido residual en tercio medio y basal; e isquemia moderada en tercio apical en el territorio de la CD. Asimismo, encontró hipocinesia moderada de la pared septal de predominio anteroseptal, a la que se le agregó diskinesia del ápex y región inferoapical durante la fase de estrés, con disminución de FEVI post estrés mayor al 5 % y dilatación transitoria del ventrículo izquierdo. 

Ante todos estos hallazgos, se decidió cirugía de revascularización miocárdica. Este procedimiento quirúrgico se realizó mediante esternotomía media, con disección de arteria radial izquierda, arteria mamaria interna (AMI) izquierda y derecha, y canulación de aorta, vena cava superior e inferior para la conexión a la bomba de circulación extracorpórea. Se anastomosó la AMI derecha a la obtusa marginal, AMI izquierda a la descendente anterior y arteria radial a la descendente posterior. Para llevar a cabo las anastomosis arteria-les se utilizaron lupas quirúrgicas de magnificación 4.5X de campo expandido (Designs for Vision, Inc.) e instrumental quirúrgico para microcirugía. Se utilizó bomba de circulación extracorpórea, hipotermia a 28ºC, solución cardiopléjica Custodiol HTK, con un tiempo de isquemia de 102 min y tiempo total de circulación extracorpórea de 126 min; sin complicaciones postquirúrgicas y buena evolución. 

A los 3 meses del postoperatorio el paciente no presenta cambios significativos en el electrocardiograma, ni en el ecocardiograma (strain global longitudinal -14% y circuferencial -13%); (Figura 3b) continúa asintomático y en tratamiento con aspirina, digoxina, clopidogrel y furosemida.

## Discusión

La enfermedad de Kawasaki tiene mayor prevalencia en Japón, con una incidencia anual de 243 casos por 100 000 niños menores de 5 años;^1^ sin embargo, en Latinoamérica se desconoce la epidemiología, carga de enfermedad, morbimortalidad y aspectos genéticos en niños de la región.^10^ Se ha reportado que el tratamiento oportuno con inmunoglobulina disminuye la sintomatología y reduce la frecuencia de compromiso coronario cuando se administra dentro de los primeros 10 días del inicio de síntomas; sin embargo, la afección coronaria es aún la complicación más grave.^11^ Kato et al.^5^ señalan que la mayoría de los infartos miocárdicos se presentan durante el primer año de la enfermedad; no obstante, la muerte súbita por infarto de miocardio puede ocurrir muchos años después en niños y adultos con aneurismas y estenosis de las arterias coronarias. De hecho, en un estudio en San Diego, entre los adultos menores de 40 años de edad con sospecha de isquemia miocárdica que se sometieron a una angiografía coronaria, aproximadamente el 5% tenía lesiones consistentes con secuelas tardías de EK.^1^

El tratamiento intervencionista con angioplastia con balón y colocación de stents es factible, pero estos no son adecuados en niños en crecimiento. Muta e Ishii^12^ informaron que el tratamiento intervencionista fue menos eficaz que la cirugía debido a la mayor necesidad de repetir los procedimientos, particularmente cuando se aplica en menores de 12 años. El primer caso de revascularización coronaria quirúrgica para la EK^13^ se realizó en 1975 con hemoconductos venosos, siendo los resultados malos; por ende, en la actualidad se realiza con hemoconductos arteriales. 

Es conocido que los aneurismas de menos de 8 mm en 50% de los casos regresan al diámetro normal de la luz interna dentro de los 2 años posteriores al inicio de la enfermedad. La posibilidad de regresión es inversamente proporcional al tamaño del aneurisma, y la regresión rara vez continúa después de 2 años.^5^ En el caso de los aneurismas gigantes (mayores de 8 mm o Z score >10), menos de la mitad disminuirá su tamaño. 

En el Instituto Nacional de Cardiología Ignacio Chávez las indicaciones de revascularización son:^9^ 1) pacientes sintomáticos con enfermedad coronaria severa que consiste en aneurismas y estenosis significativa (> 50%) y 2) pacientes asintomáticos que tienen progresión angiográfica de estenosis coronaria. La revascularización quirúrgica debe considerarse cuando hay evidencia de isquemia moderada a severa reversible, preferiblemente antes de que se presente infarto del miocardio o isquemia que condicione falla ventricular.^14^ Yamauchi et al.^15^ en un análisis multivariado establecieron que el único predictor de riesgo para el deterioro de la función ventricular fue el rango de tiempo entre la detección de enfermedad aguda y el momento quirúrgico. 

Si bien es cierto que la cirugía de revascularización coronaria fue descrita en pacientes adultos, existen de manera creciente publicaciones de series de pacientes menores de 10 años, como el caso que se presenta. La indicación de la cirugía fue el alto riesgo de la enfermedad (aneurisma gigante e isquemia moderada a severa del miocardio con tejido viable, reversible al esfuerzo comprobada por estudios de strain ecocardiográfico y medicina nuclear), pero el resultado postquirúrgico fue óptimo, con la consiguiente modificación de la evolución natural de la enfermedad y pronóstico de vida para el paciente, así como la mejora en la calidad de vida. 

Tras la evidencia de la seguridad y eficacia de este procedimiento en pacientes lactantes mayores y niños menores de 10 años,^13^ y el impacto de su pronta realización en este tipo de pacientes, las operaciones de injerto de derivación de la arteria coronaria deberían representar una parte fundamental de la capacitación de los cirujanos cardiovasculares pediátricos contemporáneos. 

## Conclusión

Actualmente, las diferentes herramientas de diagnóstico han permitido una vigilancia estrecha de los pacientes con EK y sus lesiones coronarias, lo que ha permitido establecer un tratamiento oportuno, disminuyendo el riesgo de complicaciones coronarias que son la principal causa de muerte en estos pacientes. Por sus buenos resultados, la cirugía de revascularización coronaria es el tratamiento idóneo en la edad pediátrica en casos de alto riesgo.

## Financiación:

Ninguna
